# Dynamics of *Staphylococcus aureus* in patients and the hospital environment in a tertiary care hospital in the Netherlands

**DOI:** 10.1186/s13756-023-01349-2

**Published:** 2023-12-20

**Authors:** Adriënne S. van der Schoor, Anne F. Voor in ’t holt, Willemien H.A. Zandijk, Marco J. Bruno, Diederik Gommers, Johannes P.C. van den Akker, Johanna M. Hendriks, Juliëtte A. Severin, Corné H.W. Klaassen, Margreet C. Vos

**Affiliations:** 1 Department of Medical Microbiology and Infectious Diseases, Erasmus MC University Medical Center, Rotterdam, The Netherlands; 2 Department of Gastroenterology and Hepatology, Erasmus MC University Medical Center, Rotterdam, The Netherlands; 3 Department of Adult Intensive Care, Erasmus MC University Medical Center, Rotterdam, The Netherlands; 4 Department of Surgery, Erasmus MC University Medical Center, Rotterdam, The Netherlands

**Keywords:** *Staphylococcus aureus*, Staphylococcal protein A, Patient’s rooms, Health Facility Environment, Surfaces

## Abstract

**Background:**

The dynamics of *Staphylococcus aureus* in patients and the hospital environment are relatively unknown. We studied these dynamics in a tertiary care hospital in the Netherlands.

**Methods:**

Nasal samples were taken from adult patients at admission and discharge. Isolates cultured from clinical samples taken before and during hospitalization from these patients were included. Environmental samples of patient rooms were taken over a three-year period. Finally, isolates from clinical samples from patients with an epidemiological link to *S. aureus* positive rooms were included. Staphylococcal protein A (*spa*) typing was performed.

**Results:**

Nasal samples were taken from 673 patients. One hundred eighteen (17.5%) were positive at admission and discharge, 15 (2.2%) patients acquired *S. aureus* during hospitalization. Nineteen patients had a positive clinical sample during hospitalization, 15.9% of the *S. aureus* were considered as from an exogenous source. One hundred and forty (2.8%) environmental samples were *S. aureus* positive. No persistent contamination of surfaces was observed. Isolates were highly diverse: *spa* typing was performed for 893 isolates, identifying 278 different *spa* types, 161 of these *spa* types were observed only once.

**Conclusion:**

Limited transmission could be identified between patients and the hospital environment, and from patient-to-patient. Exogenous acquisition was assumed to occur in 15% of clinical samples. Environmental contamination was infrequent, temporarily, and coincided with the strain from the patient admitted to the room at that time. MRSA was rare and not found in the environment.

**Supplementary Information:**

The online version contains supplementary material available at 10.1186/s13756-023-01349-2.

## Introduction

Healthcare-associated infections (HAI) are a worldwide problem, lengthening hospital stay, morbidity, and mortality in affected patients; all considerably increasing healthcare costs. One of the leading bacteria causing HAI is *Staphylococcus aureus*. *S. aureus* is an opportunistic pathogen that colonizes the nose and skin, but can cause a range of infections, e.g., skin and surgical site infections [1]. Additionally, *S. aureus* is an important cause of both community- and hospital-acquired bacteraemia, with a mortality rate between 15 and 25% [2, 3]. Nasal carriage of *S. aureus* is a risk factor for acquiring HAI [4]. Approximately one third of the population is a carrier of methicillin-susceptible *S. aureus* (MSSA), however, the prevalence of methicillin-resistant *S. aureus* (MRSA) carriers in the Netherlands is much lower, 0.03–0.17% [5, 6].

Of all *S. aureus* infections, 80% are endogenous [7, 8]. Hence, it is estimated that only 20% of *S. aureus* infections is exogenous. The latter patient group, although not well understood, tend to have longer hospitalizations following bacteraemia and a higher risk of mortality compared to patients with endogenous infections [7]. Consequently, preventing acquisition of *S. aureus* in the hospital is essential. Acquisition can occur via contact with colonized patients or personnel, but also via direct or indirect contact with contaminated surfaces [9].

While the clinical relevance of *S. aureus* and the ability to contaminate surfaces are known, the dynamics within the hospital environment and between the hospital environment and patients are relatively unknown. Therefore, we aimed to determine these dynamics within our hospital by examining carriage and acquisition of *S. aureus* in patients, and to determine environmental contamination rates by *S. aureus*. Finally, we aimed to identify transmissions between patients and the environment.

## Methods

### Setting and study design

This study was performed at the Erasmus MC University Medical Center, in Rotterdam, the Netherlands (Erasmus MC). In May 2018, the Erasmus MC relocated from an old, 1125-bed hospital with mainly multiple-occupancy rooms to a newly constructed, 525-bed hospital building with 100% single-occupancy rooms and private bathrooms. Between 2018 and 2022, approximately 30,000 patients are admitted yearly, with an average length of stay of 6 days.

**Fig. 1:**
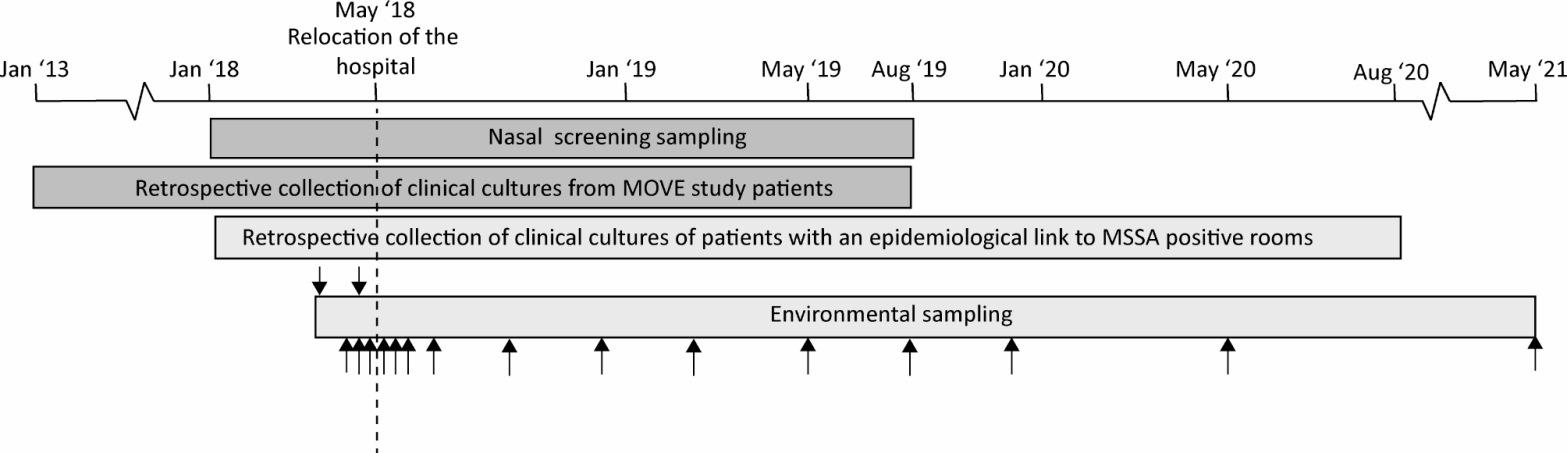
Timeline of nasal screening sampling, environmental sampling, and retrospective collection of *S. aureus* positive clinical cultures. Included patients refers to patients who participated in nasal sampling. Arrows indicate the 17 sampling moments of the environment, the two arrows above indicate sampling moments in the old building, arrows below indicate sampling moments in the new building. Dark grey indicates the timeline for samples related to included patients, light grey indicates the timeline for samples related to the hospital environment

This observational study was part of the MOVE study, which was designed to determine the effect of the relocation to a hospital with 100% single-occupancy rooms on microbial safety [10, 11]. For this study, patients were sampled at admission and discharge to determine the presence of MSSA and MDRO between January 1, 2018 and August 31, 2019 (Fig. 1). Additionally, environmental samples were taken to determine environmental contamination between April 2018 and May 2021 (Fig. 1). Participating departments were the adult intensive care unit (ICU), cardiology, gastroenterology and hepatology, general surgery, hematology, internal medicine, nephrology, neurology, neurosurgery, orthopedics, and plastic surgery, in both hospital buildings. These selected departments represent a large part of the patient population of the Erasmus MC, with the exception of some smaller departments such as pulmonology, dermatology, and ear nose and throat.

The study described in this manuscript includes the prospective screening of patients and the environment. Additionally, *S. aureus* positive clinical cultures were retrospectively collected from January 2013 until August 2020.

### Data collection

*S. aureus* isolates identified from three sample types were included: (1) nasal screening samples taken from included patients, (2) clinical samples taken from selected patients admitted to our hospital, and (3) environmental samples.

#### Nasal samples

All patients admitted to participating wards between January 1, 2018 and August 31, 2019, were approached for participation as part of the MOVE study (Fig. 1). Inclusion criteria were ≥ 18 years and an expected hospital stay of ≥ 48 h. After informed consent, nasal swabs were taken with flocked e-swabs (FLOQSwabs®; Copan, Italy) within 24 h of admission and on the day of discharge. Patients missed at discharge were requested by mail to take the swab at home. Nasal samples taken between January 2018 and June 2018 were stored in -80 °C after 0.3 mL 99.5% glycerol was added. From June 2018 onwards, samples were processed directly.

#### Clinical cultures

Clinical cultures are cultures that are taken as part of standard patient care. A positive clinical culture is included as such, and therefore does not necessarily indicate infection. We did not check patients’ condition or clinical state or use definitions for infections. Isolates from all types of cultures (e.g. blood, liquor, skin) were included from patients from whom admission and discharge cultures were taken for the MOVE study. Additionally, isolates from patients with an epidemiological link to a positive patient room were included (Fig. 1). Patients had an epidemiological link to the room when they were admitted to the ward of the positive room within three months prior to or after environmental sampling and when they had an *S. aureus* positive clinical culture. The epidemiological links were determined for all patients admitted to our hospital, including the patients included in the MOVE study. .Epidemiological links were determined for *S. aureus* positive rooms between April 2018 and May 2020. Per patient, one MSSA and/or MRSA isolate per *spa-*type was included. To determine if clinical isolates were identical to the isolates identified in the nasal screening cultures, we retrospectively determined if patients included in the MOVE study had For *S. aureus* positive clinical cultures taken between 2013 and the start of their hospitalization, and/or cultures taken during their hospitalization (Fig. 1). These isolates were also typed.

#### Environmental samples

Environmental samples were taken 17 times at different intervals from April 2018 till May 2021, in both the old and the new hospital building (Fig. 1) [11]. In the old building, samples were taken two times, in the new building, samples were taken 15 times, from two weeks before to 36 months after relocation (Fig. 1) [11]. At one time 14 sites were sampled per (bath)room and 40 (bath)rooms in the old and 42 in the new building were sampled. In the old building, 10 two-person rooms, 15 four-person rooms, four isolation rooms with anteroom, three hematology rooms with anteroom, 10 ICU rooms, of which two with anteroom, and nine bathrooms were sampled. In the new building, 23 rooms were sampled on surgical and medical departments, four isolation rooms with an ante, three haematological rooms with an ante, 10 ICU rooms, and 10 bathrooms. The bathrooms belonged to eight sampled rooms on the surgical and medical departments, one isolation room and one haematological room [11]. Different locations in patient rooms and bathrooms were sampled, such as the nightstand, the sink, and the shower chair (Supplementary file 1) [11]. Samples were taken with cotton swabs (BSN medical, Almere, the Netherlands), pre-moistened with PBS. Additional information about sampling methods is described in van der Schoor et al. [11].

### Microbiological methods

For nasal samples, 800µL swab medium was pipetted in a 6.5% NaCl TSB and incubated for 24 h at 35 °C. For environmental samples, swabs were placed in a 75 mg/L aztreonam TSB directly after sampling, and incubated overnight at 35 °C. After incubation, nasal and environmental samples were processed as follows: A PCR was performed to identify the *S. aureus nucA-* and *mecA*/*mecC* genes (Supplementary file 2). When *S. aureus* was identified, a blood agar was inoculated and incubated twice overnight at 35 °C. The MALDI-TOF Biotyper (Matrix Assisted Laser Desorption Ionization-Time of Flight, Bruker Daltonics, Bremen, Germany) was used for the identification of all morphologically different suspected colonies. To determine beta-lactam antibiotic resistance, a cefoxitin disk diffusion test was performed. A growth inhibition zone of < 22 mm after 18–24 h was considered resistant [12]. Isolates were stored at -80 °C. Clinical samples were processed according to routine diagnostic protocols as above.

#### Staphylococcal protein a typing

All included isolates were analysed by *spa*-typing using established procedures [13]. Simpson’s index of diversity was calculated separately for the MOVE study nasal strains, clinical strains, and environmental strains [14]. In the calculations, one isolate per patient per *spa* type was included for the nasal strains, as well for the clinical strains. For the environmental isolates, one isolate per *spa* type per room per sampling moment was included in the calculation.

### Definitions

Colonization was defined as having a nasal screening sample with *S. aureus* at both admission and discharge, with both *S. aureus* being of the same spa type. Acquisition was defined as a negative culture at admission and a positive *S. aureus* culture at discharge, or when the discharge isolate was not identical to the admission strain. Loss of nasal carriage was defined as a positive culture at admission and a negative culture at discharge. When the clinical isolate was identical to the nasal isolate, the clinical isolate was considered endogenous. When the clinical isolate was not identical to the nasal isolate, the clinical isolate was considered acquired/exogenous.

### Statistical analyses

Descriptive analyses were performed. For continuous variables, medians with range are presented. Normal distributed variables were analysed with independent sample t-tests. Categorical variables are presented as percentages and analysed using a Chi-squared test. *P-*values < 0.05 were considered statistically significant. For all analyses IBM Statistical Package for the Social Sciences Solutions (SPSS) version 25 (IBM Corp., Armonk, New York, USA) was used.

## Ethical approval

This study was approved by the medical ethical research committee of the Erasmus MC (MEC-2017-1011), and was not subject to the Medical Research Involving Human Subjects Act. Written informed consent was obtained from patients included in the MOVE study. Passive informed consent was accepted for patients admitted to the ICU and for patients who did not participate in the MOVE study, but from whom clinical cultures were included. Patients who did not allow that their data were to be used for research were not included in the study population. This study was registered in the Dutch National Trial Register (NL8406) in February 2020.

## Results

### Nasal screening samples

**Fig. 2:**
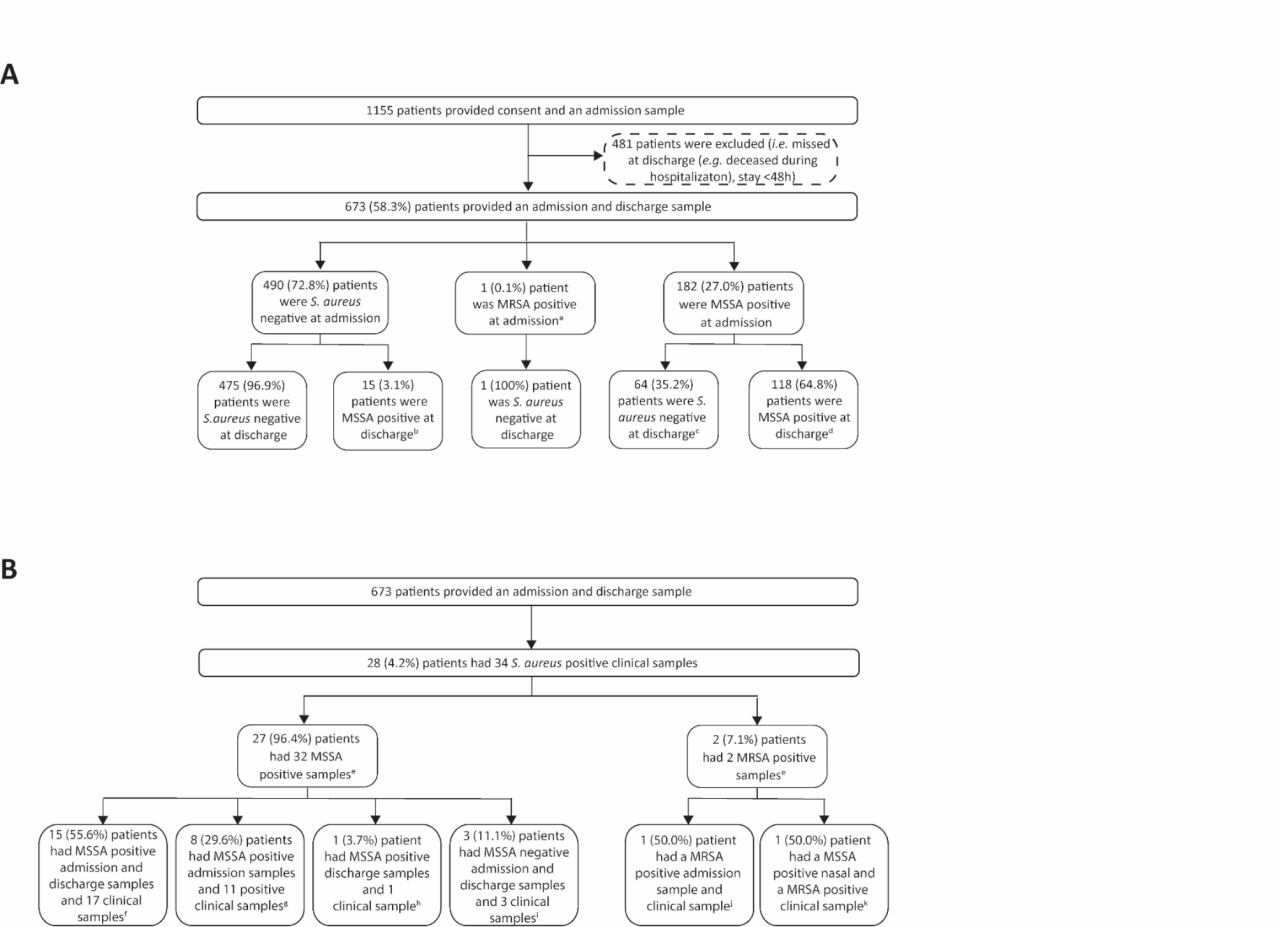
**(A)** Flowchart of prospective patient inclusion and sample results **(B)** Flowchart of clinical samples and the relation to the results of nasal samples ^a^ Spa typing was performed on the MRSA isolate ^b^ 14 (93.3%) isolates were typed ^c^ 62 (96.9%) of isolates were typed ^d^ 109 (92.4%) admission isolates were typed, 115 (97.5%) discharge isolates were typed, 106 (89.8%) complete sets were typed ^e^ 1 patient had both a clinical MSSA and MRSA ^f^ 30 (100%) nasal isolates were typed and 17 (100%) clinical isolates were typed ^g^ 6 (75.0%) nasal isolates and 11(100%) clinical isolates were typed ^h^ 1 (100%) nasal isolates and 1 (100%) clinical isolate was typed ^i^ 3 (100%) clinical isolates were typed ^j^ 1 (100%) clinical MRSA isolate was typed ^k^ 1 (100%) nasal isolate was typed and 1 (100%) MRSA isolate was typed

Admission and discharge nasal samples were taken from 673 patients (Fig. 2a). Three hundred fifteen (46.8%) patients were female, the median duration of hospital stay was six days (range: 2-146 days). In total, 197 (29.2%) patients had ≥ 1 MSSA positive nasal sample (79 (40.1%) patients had one positive sample and 118 (59.9%) had two positive samples), and one (0.1%) patient was positive for MRSA upon admission (Fig. 2a). Twenty-one patients were hospitalized multiple times during the study period; 18 patients twice and three patients three times. No significant differences between the period in the old and the new hospital building were found in the number of MSSA positive patients at admission, positive at discharge, for acquisition and for loss (data not shown). Therefore, data from both buildings were combined.

**Fig. 3:**
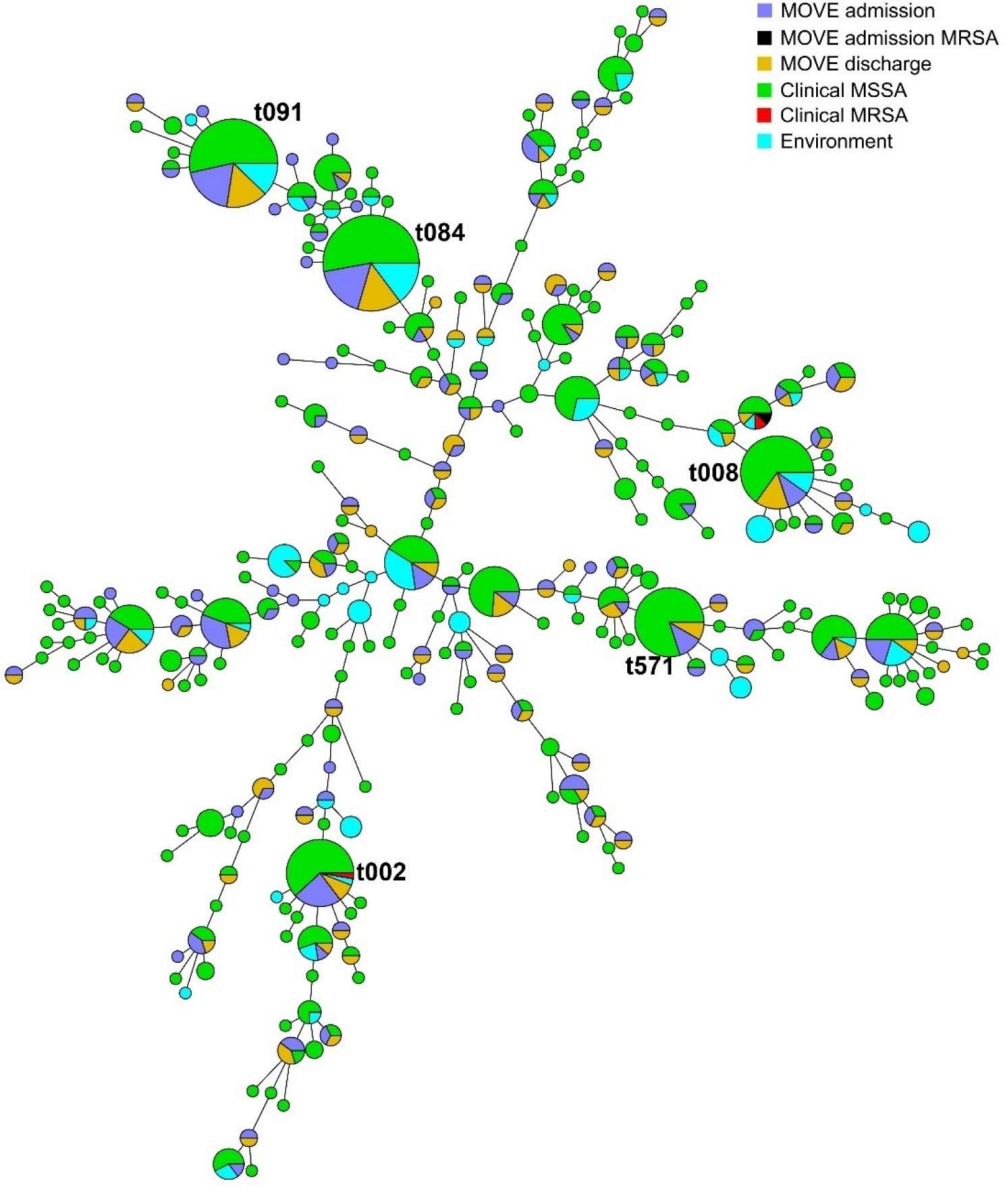
Minimum spanning tree of the 893 typed *S. aureus* isolates created using BioNumerics v7.6 using default settings. Each circle indicates a *spa* type, 278 *spa* types were observed, 161 *spa* types were observed only once. The size of the circles corresponds to the number of isolates with that *spa* type. The five most prevalent spa types are indicated. Colours indicate the origin of the isolate

In total, 161/182 (88.5%) admission isolates and 125/133 (94.0%) discharge isolates were available for *spa* typing (Fig. 3). The 286 isolates belonged to 110 different strain types, of which 45 *spa* types (40.9%) were observed once. The Simpson’s diversity index was 0.984 for admission isolates and 0.986 for discharge isolates. The most prevalent *spa* types were t084 (N = 22, 8.1%), t091 (N = 20, 7.3%), and t002 (N = 11, 4.2%) (Supplementary file 3). From 106 patients, both the admission and discharge strain were typed. Ninety out of 106 (84.9%) of patients were colonized with *S. aureus* (i.e., the same admission and discharge *spa*-type) and 16 (15.1%) patients acquired *S. aureus*. From the 15 patients who acquired MSSA during hospitalization, 14 isolates were typed. These 14 isolates belonged to 13 different *spa* types, of which 12 observed once. All typing results can be found in Supplementary file 3.

### Clinical samples

#### MSSA

Five hundred MSSA isolates from clinical samples from 487 patients were available for *spa* typing (Fig. 3). Most isolates were identified from the nose (n = 118), sputum (n = 103) or blood (n = 50). Thirty-two isolates (6.4%) were identified before or during hospitalization from patients included in the MOVE-study (Fig. 2b). Four-hundred and sixty-eight (93.4%) isolates from 418 patients were included due to an epidemiological link with a contaminated room. Two hundred and fifteen different *spa* types were detected, 159 (74.0%) types were observed once (diversity index 0.9799). The most prevalent *spa* types were t084 (N = 36, 7.2%), t091 (N = 31, 6.2%), and t571 (N = 28, 5.6%) (Supplementary file 3).

#### MRSA

Two MRSA isolates were identified from patients included in the MOVE study. The two isolates belonged to *spa* type t304 and t002 (Fig. 3, Supplementary file 3). As no MRSA was found in the environment, per definition no epidemiological link with a patient could be established.

### Comparing nasal and clinical samples of the same patients

Twenty-eight (4.2%) out of 673 patients had clinical samples positive for *S. aureus* taken before or during their hospitalization, 26 patients were MSSA positive, one patient MRSA positive, and one patient was MSSA and MRSA positive (Fig. 2b). For the MRSA positive patient, the clinical sample was taken during hospitalization. This patient was also positive for MRSA upon admission to the hospital, both isolates belonged to *spa* t304. From the 27 MSSA positive patients, 32 MSSA isolates were cultured. For 22 clinical isolates, belonging to 22 patients, the clinical isolate was identical to the admission or discharge isolate. For 16 (72.7%) isolates the sample was taken during hospitalization, for six (27.3%) isolates the sample was taken < 1 year before hospitalization. For ten (31.3%) isolates from eight patients, the clinical isolate was not identical to the admission or discharge nasal isolate. Of these ten, three (30.0%) isolates were identified from a clinical sample taken during the hospitalization, for two (20.0%) this was < 1 year before hospitalization and for five (50.0%) this was > 1 year before hospitalization. By definition, three of the 19 (15.9%) isolates identified in clinical cultures during the hospitalization had an exogenous source.

### Presence of *S. aureus* in the environment

**Fig. 4:**
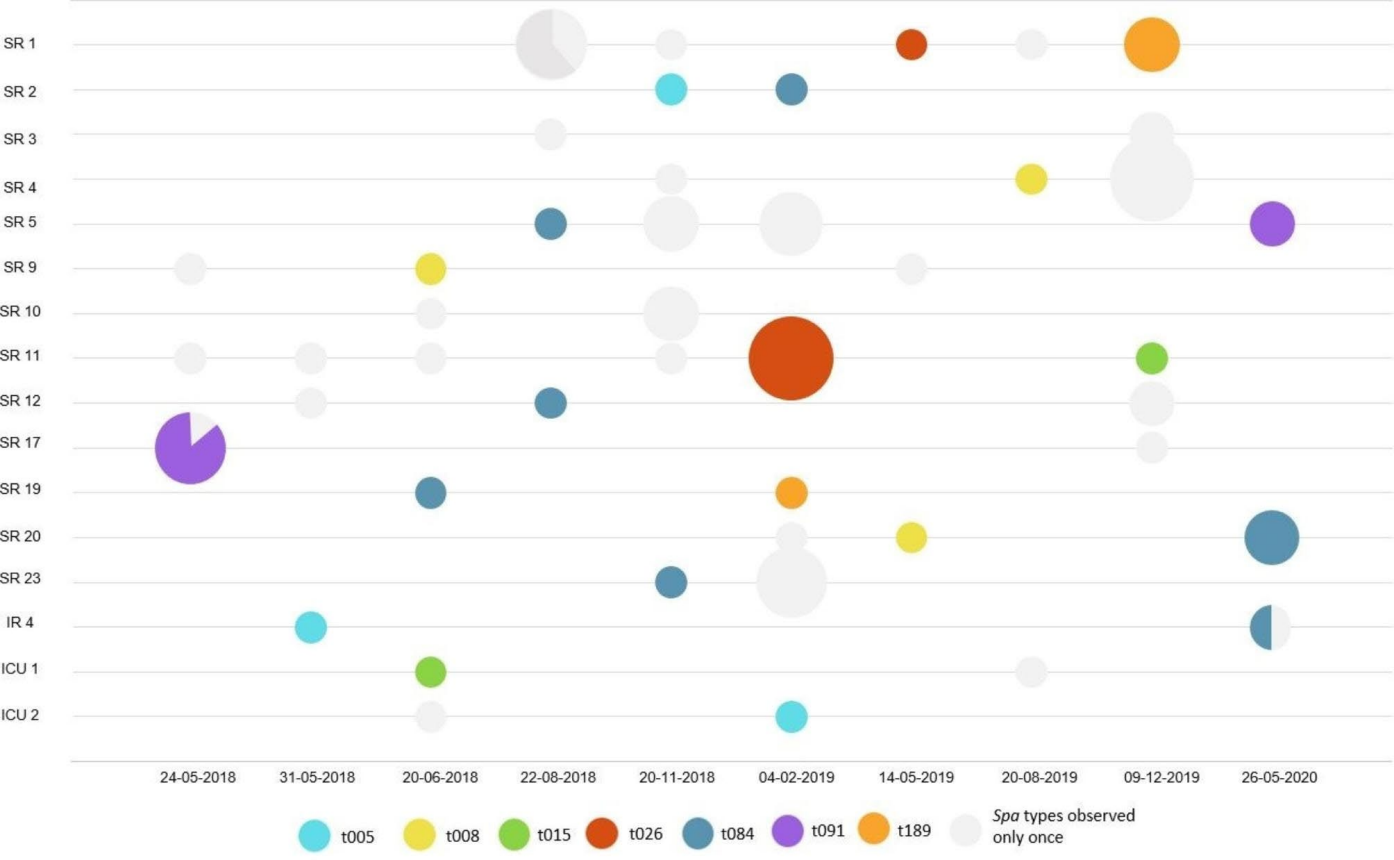
*Spa* types found over time in rooms that were positive at more than one sampling moment. *Spa* types observed at different moments or in different rooms are colored, *spa* types observed at only one moment in one room are grey. Darker grey was used to indicate that two *spa* types were identified at one moment, but in the same room. Node sizes are relative to the number of MSSA strains found. Abbreviations: SR single-occupancy room, IR Isolation room, ICU Intensive Care Unit

In total, 4,993 environmental samples were taken, 724 (14.5%) in the old and 4,269 (85.5%) in the new hospital building. No MRSA was detected, MSSA was found on 22/724 (3.0%) surfaces in the old building, compared to 120/4269 (2.8%) surfaces in the new building (*P* = 0.733) (Supplementary file 4). The rate of contaminated surfaces in the old building was between 1.6% and 4.4%, and in the new building between 0.5% and 8.5% (Supplementary file 4). In the old building, 11 out of 42 rooms (26.2%) and 2 out of 9 bathrooms (22.2%) had at least one positive location for MSSA during the two sampling moments. In the new building, 31 out of 40 rooms and bathrooms (77.5%) had at least one location positive for MSSA over the three year follow up. Of these 31 rooms, 16 were positive at multiple sampling moments over the three year period (Fig. 4). One hundred and forty-five MSSA isolates were identified on 142 surfaces, 104 isolates were available for *spa* typing, all from the new hospital building. Forty-five different *spa* types were detected, 24 (54.5%) were observed once (diversity index 0.9773). Twenty-eight (26.9%) isolates belonged to 14 (31.1%) *spa* types that were only observed in the environment. The most prevalent *spa* types were t084 (N = 10, 9.6%), t026 (N = 8, 7.7%), t091 (n = 7, 6.7%), and t7384 (N = 7, 6.7%) (Supplementary file 3). When multiple locations (n = 45) in one room were simultaneously positive, the isolates were of the same *spa* type, with three (6.7%) exceptions (Fig. 4). In all cases when positive cultures were taken from the same site over time, isolates were of different *spa* types, indicating no long-term contamination (Fig. 4). No surfaces positive at both sampling moments were identified in the old hospital building.

### Epidemiological link between clinical strains and environmental strains

Of the 468 typed clinical isolates from patients with an epidemiological link to the ward of a positive room, 16 (3.4%) isolates of 16 patients were of the same spa type as the environmental MSSA isolate. For eight patients (50.0%), the clinical sample was taken before the environmental sample was taken (3 to 62 days earlier), for eight (50.0%) patients, cultures were taken after environmental sampling (7 to 75 days after). Seven patients were admitted to a room that was found to be contaminated by environmental sampling; four (25.0%) patients during environmental sampling, and were most likely the source of the environmental contamination. Two (12.5%) patients were admitted to a positive room and discharged before environmental sampling was performed, and were the likely source of the contamination. One (6.3%) patient was admitted to a positive room 61 days after environmental sampling. Nine patients were admitted to the ward in another room than the contaminated room, therefore for these patients no transmission route could be defined .

## Discussion

In this extensive study of *S. aureus* from patients and the environment in a large tertiary care hospital in the Netherlands, we found that isolates were highly diverse and were, with exclusion of one MRSA strain, exclusively MSSA. We described the dynamics of *S. aureus* during hospitalization and our results indicate an exogenous source for the isolate in 15.9% of patients after comparing nasal screening isolates to clinical isolates. Environmental contamination was rare and temporarily and when found most likely caused by the patient admitted to the room at the time of sampling. Our results show that transmission most frequently occurred from the patient to the environment.

We showed that almost 1/6th of patients with a positive nasal sample and a clinical sample taken during hospitalization had a possible exogenous source of *S. aureus.* Fifteen patients acquired *S. aureus* isolates in their nose, indicating an exogenous source. While it is possible that some patients had a false-negative admission culture or were colonized at another body site (approximately 6% of MSSA carriers [15]), it is unlikely that this was the case for all patients. Patients could have carried multiple *S. aureus* types simultaneously. Both Wertheim et al. and Cespedes et al. have shown that ~ 6.6–10% of *S. aureus* carriers can carry multiple *S. aureus* types simultaneously [6, 16]. Although we analysed all morphologically different MSSA isolates, it is likely that multiple *S. aureus* strain types have been missed.

Our results showed that the patient is the most likely source for environmental contamination and not vice versa. We did not detect any long-term presence of *S. aureus* in the old or new hospital building. The low contamination rate indicates that our cleaning protocols are effective in removing MSSA from contaminated surfaces. Our cleaning protocol consists of daily cleaning with dampened microfiber cloths, without added cleaning- or disinfection solution, unless disinfection is indicated (e.g. after discharge of a MRSA carrier). In the new hospital building, a final cleaning after discharge of the patient was introduced. However, given the lack of long-term presence of *S. aureus* in the old building, our cleaning protocol was most likely already effective for MSSA before introducing the final cleaning step. Another possibility is that *S. aureus* strains normally does not survive in the environment. Our sampling method could also have impacted the recovery rate of *S. aureus*. Sampling with cottons swabs has several advantages, such as the ability to sample all different types of surfaces [17]. Nonetheless, recovery rates for *S. aureus* are low, and due to the difficulty in standardization of sampling, recovery rates in vitro range between 22 and 58% [17, 18]. Using contact plates could have resulted in higher recovery rates [19–21]. However, this method also has disadvantages, such as difficulty sampling specifically shaped surfaces (e.g., door handles), and with this method we could not use selective broths which helped us detect *S. aureus* in low abundances. Another explanation is that we were unable to detect *S. aureus* due to dry biofilm formation [22]. Multiple studies have shown that dry biofilms can be present on most sampled surfaces. Viable bacteria were identified in biofilms, although no planktonic bacteria were present on the surfaces [23–25]. Hu et al. showed that over 90% of ICU surfaces contained bacteria in biofilms, and that *S. aureus* was present in 50% of the cultures [25]. Additionally, biofilm hampers cleaning and disinfection [25, 26]. Consequently, the low contamination rates we found could be an underestimation, although we found no indication for transmission to patients.

It is important to note that the last two environmental sampling moments, in May 2020 and May 2021, took place during the COVID-19 pandemic. Consequently, these sampling moments might not reflect the expected contamination rates in a non-pandemic setting, as a result of the use of facemasks and improved hand hygiene. However, the contamination rates in May 2020 were comparable to earlier sampling moments. In May 2021, the environmental contamination was comparable to the contamination before opening the hospital and was most likely influenced by the pandemic. This was an important factor not to determine transmission from patients to the environment and vice versa for this last sampling moment.

The identified *S. aureus* population was highly diverse. While some clusters were identified, many *spa* types were only observed once. The low number of identified MRSA isolates was as expected, given the low prevalence rates in the Netherlands. The low observed transmission from and to the hospital environment is supported by the fact that a number of environmental isolates belonged to *spa* types only observed once. As the study was not set up to include all patients admitted to the sampled patient rooms, this is not unsurprising. Remarkably, 26.9% of environmental *S. aureus* isolates belonged to a *spa* type not identified in nasal or clinical strains pointing to personnel as likely source of these isolates.

### Strengths and limitations

The main strength of this study was that we looked at the dynamics in nasal carriage, clinical samples, and the environment over a three-year period. Additionally, sampling over a three-year period enabled us to determine long-term presence of *S. aureus.* Our study also has several limitations. First, and most important, we did not include all patients admitted to the sampled rooms, especially around and during sampling moments. Subsequently, we were limited to determine transmissions to the environment through clinical samples. Consequently, our results most likely show an underestimation of transmissions. Second, our results likely show an underestimation of the environmental contamination rates, and consequently transmissions, due to the limitations of the sampling method and the possible presence of dry biofilm. Third, we performed *spa* typing, instead of whole genome sequencing (WGS), which may overestimate relatedness between isolates. While the discriminatory power of WGS is higher, for the purpose of our study, we believe the discriminatory power of *spa* typing was sufficient since the diversity index for all sample types was well over > 0.95, the criterion described by van Belkum et al. [27]. Fourth, we did not determine antibiotic usage, which could partially explain dynamics within patients. Moreover, we did not include healthcare workers, who are a known reservoir of *S. aureus.* Including healthcare workers would have increased identifying the source of strains that were only identified in the hospital environment, or potentially of strains acquired by patients. However, since our study was mainly focused on transsmission from patients to the hospital environment, we indirectly included healthcare workers as a source. Finally, we only sampled patients once at admission and once at discharge. Additionally, we only sampled the nose and not other body sites, such as the oral cavity [21, 28]. Both these factors could have lead to an underestimation of the number of carriers. However, studies have shown that two nasal swabs taken with a week interval can classify MSSA nasal carriage accurately [29]. Furthermore, they found persistent carriers did not show one positive and one negative sample in this order taken one week apart. Therefore, it is unlikely that we missed persistent carriers.

## Conclusion

Our results show that environmental contamination was rare, with no long-term contamination of surfaces. The dynamics in environmental contamination by S aureus is highly influenced by the admitted patients, and therefore highly variable, and assumed to be continuously changing. We considered an exogenous source for almost one sixth of patients, which is congruent with literature. To optimise detecting contamination of the environment, future research should focus on the role of dry biofilms and on methods of sampling and culture. Last, we performed our study in a low endemic setting for MRSA, which does not allow extrapolation of our results to a high endemic setting.

### Electronic supplementary material

Below is the link to the electronic supplementary material.

Supplementary Material 1: Sampled locations.

Supplementary Material 2: *Staphylococcus aureus* PCR.

Supplementary Material 3: Prevalence of identified *spa* types.

Supplementary Material 4: Positive *Staphylococcus aureus* locations.

## Data Availability

Patient data from this study are available upon request because there are ethical restrictions on sharing the data publicly. Approval to conduct the study was received from the medical ethical research committee of the Erasmus MC (MEC-2017-1011) (Dr. Molewaterplein 40, 3015 GD Rotterdam, Room Ae-337, T: +31 (0)10–70 34428, E: metc@erasmusmc.nl). Data requests can be sent to the department of Medical Microbiology and Infectious Diseases of the Erasmus MC (E: info.microbiologie.infectieziekten@erasmusmc.nl).

## Abbreviations

Erasmus MC: Erasmus MC University Medical Center
HAI: Healthcare-associated infections
ICU: Intensive care unit
IR: Isolation room
MALDI-TOF: Matrix Assisted Laser Desorption Ionization-Time of Flight
MSSA: Methicillin-susceptible *S. aureus*
MRSA: Methicillin-resistant *S. aureus*
Spa: Staphylococcal protein A
SR: Single-occupancy room
TSB: Tryptic soy broth
