# Supplementary material for: Dynamics of Staphylococcus aureus in patients and the hospital environment in a tertiary care hospital in the Netherlands
Source: Antimicrob Resist Infect Control. 2023 Dec 20;12:148. doi: 10.1186/s13756-023-01349-2 (PMC10734193; doi:10.1186/s13756-023-01349-2)

**Supplementary file 1.** Sampled locations

**Supplementary figure 1.1.** Floorplan of two- and four patient room in the old hospital building with sampled locations. Additional sampling locations for four patient rooms are indicated in grey.


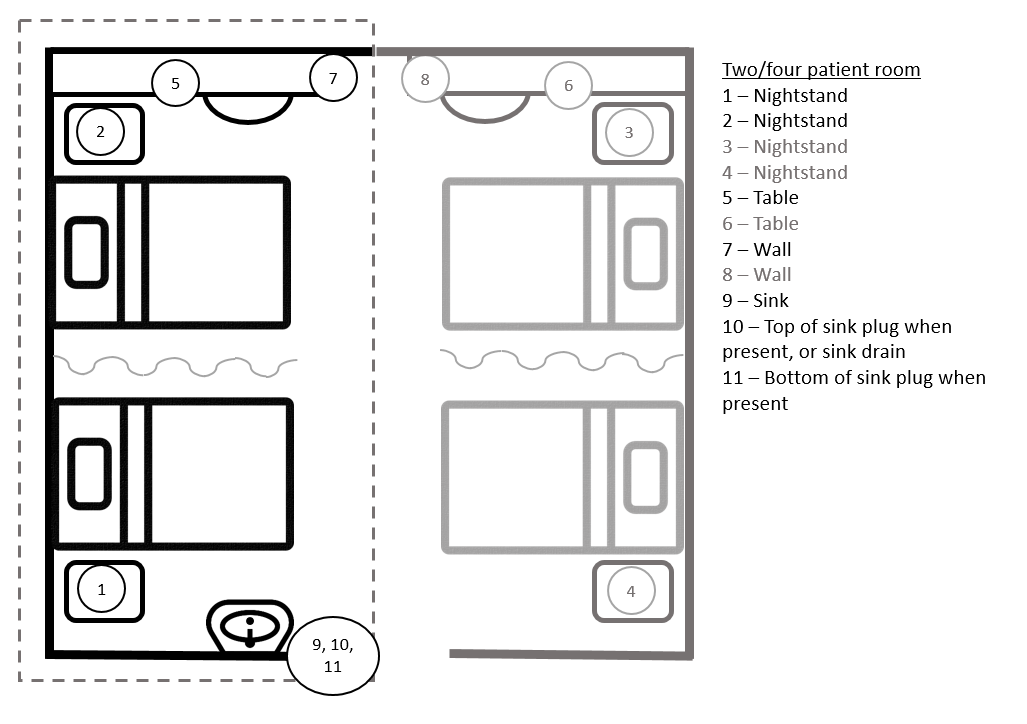


**Supplementary figure 1.2.** Floorplan of bathrooms in the old hospital building, with sampled locations


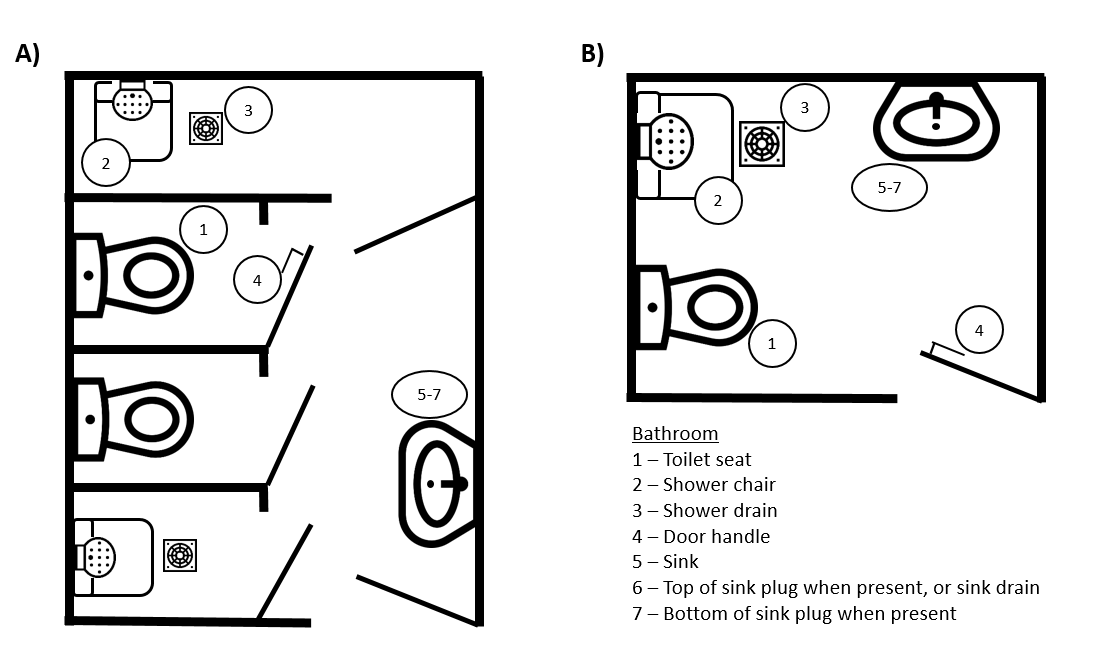


**Supplementary figure 1.3.** Floorplan of single-occupancy room in the new building, with sampled locations. The light grey line indicates where the door of the ante room is located, when an ante room is present.


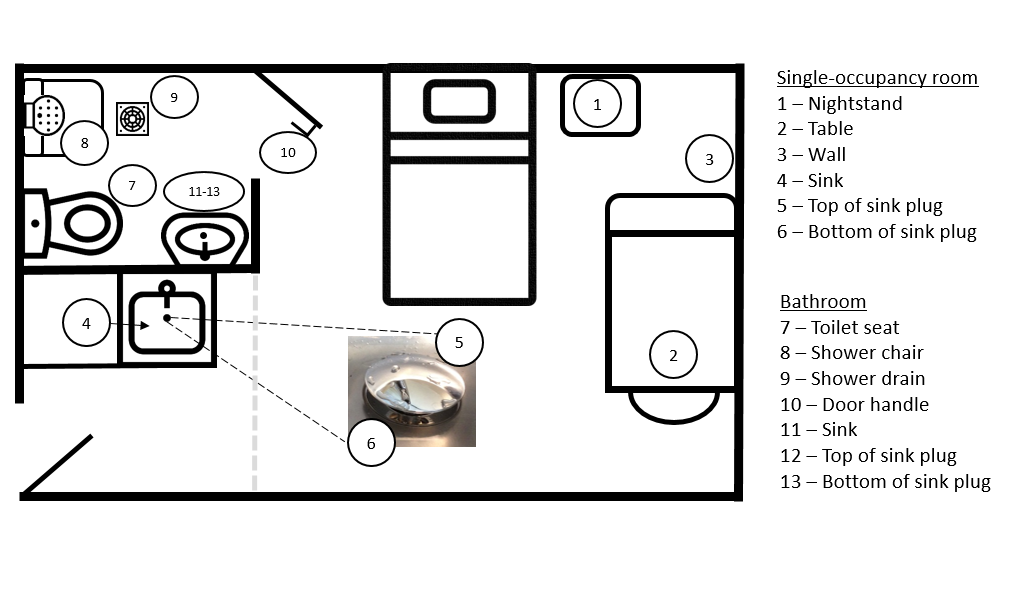

Supplement: Supplementary file 1 — Supplementary Material 1: Sampled locations. [file 13756_2023_1349_MOESM1_ESM.docx]
