# Supplementary material for: Dynamics of Staphylococcus aureus in patients and the hospital environment in a tertiary care hospital in the Netherlands
Source: Antimicrob Resist Infect Control. 2023 Dec 20;12:148. doi: 10.1186/s13756-023-01349-2 (PMC10734193; doi:10.1186/s13756-023-01349-2)
Supplement: Supplementary file 2 — Supplementary Material 2: Staphylococcus aureus PCR. [file 13756_2023_1349_MOESM2_ESM.docx]

**Supplementary file 2. *S. aureus* PCR**

DNA was isolated from freshly grown samples using the MagNA Pure 96 platform in combination with the MagNA Pure 96 DNA and Viral Nucleic Acid Small Volume Kit (Roche Diagnostics, Almere, the Netherlands) as recommended by the manufacturer. Prior to extraction, samples were spiked with Phocine Herpesvirus (PhHV) (Viroscience, Erasmus University Medical Center MC, Rotterdam, the Netherlands) as an internal process control. A multiplex real-time PCR was performed on the LightCycler 480 platform (Roche Diagnostics) with maximum heating and cooling settings. Amplification reactions (20µL) consisted of 5 µL DNA, primers and probes (sequences and concentrations according to Table 1) in 1x LightCycler 480 Probes Master (Roche Diagnostics). Cycling parameters involved an initial denaturation for 5 min at 95°C followed by 50 cycles of 95°C for 5s and 60°C for 30s after which the samples were cooled down.

**Supplementary table 1.** Primers and probes used in the PCR screening assay

| Target |  | Conc. (µM) | (reporter label)-sequence-(quencher) |
| --- | --- | --- | --- |
| *nucA* (*S. aureus*) | Forward | 0.5 | TGCTGATGGAAAAATGGTAAAC |
|  | Reverse | 0.5 | AAAWGTTGTTCATGTGTATTGTTAGG |
|  | Probe | 0.1 | (Cy5)-TCGTCAAGGCTTGGCTAAAGTTGCT-(BHQ2) |
| *mecA* | Forward | 0.5 | AACTTAATTGGCAAATCCGGTA |
|  | Reverse | 0.5 | AAACCACCCAATTTGTCTGC |
|  | Probe | 0.1 | (FAM)-CTGCAGAACTCAAAATGAAACAAGGAGAAA-(EDQ) |
| *mecC* | Forward | 0.5 | CGCATTGCATTAGCATTAGG |
|  | Reverse | 0.5 | AAAAGGGATAATCACTCGGGATA |
|  | Probe | 0.1 | (TR)-TGCAAGATTTGGGAATCGGTGAAAA-(BHQ2) |
| PhHV | Forward | 0.5 | GGGCGAATCACAGATTGAATC |
|  | Reverse | 0.5 | GCGGTTCCAAACGTACCAA |
|  | Probe | 0.1 | (YY)-TTTTTATGTGTCCGCCACCATCTGGATC-(EDQ) |
