# Supplementary material for: Dynamics of Staphylococcus aureus in patients and the hospital environment in a tertiary care hospital in the Netherlands
Source: Antimicrob Resist Infect Control. 2023 Dec 20;12:148. doi: 10.1186/s13756-023-01349-2 (PMC10734193; doi:10.1186/s13756-023-01349-2)
Supplement: Supplementary file 3 — Supplementary Material 3: Prevalence of identified spa types. [file 13756_2023_1349_MOESM3_ESM.docx]

**Supplementary file 3.** Prevalence of spa types identified in nasal samples, clinical samples and environmental samples

^a^acquired strains are also included in discharge strains.

Abbreviations: MSSA methicillin-susceptible *Staphylococcus aureus*, MRSA methicillin-resistant *Staphylococcus aureus*

| ***Spa*-**  **type** | **Nasal samples (N=286)** | **Admission (N=161)** | **Admission MRSA (N=1)** | **Discharge (N=125)** | **Acquired (N=14)^a^** | **Clinical MSSA (N=500)** | **Clinical MRSA (N=2)** | **Environment (N=104)** | **Total (N=983)** |
| --- | --- | --- | --- | --- | --- | --- | --- | --- | --- |
| t084 | 22 | 12 | 0 | 10 | 1 | 36 | 0 | 10 | 68 |
| t091 | 20 | 11 | 0 | 9 | 2 | 31 | 0 | 7 | 58 |
| t008 | 10 | 4 | 0 | 6 | 1 | 26 | 0 | 4 | 40 |
| t571 | 7 | 4 | 0 | 3 | 0 | 28 | 0 | 0 | 35 |
| t002 | 11 | 8 | 0 | 3 | 0 | 21 | 1 | 1 | 34 |
| t026 | 5 | 3 | 0 | 2 | 0 | 9 | 0 | 8 | 22 |
| t012 | 6 | 4 | 0 | 2 | 0 | 10 | 0 | 4 | 20 |
| t230 | 5 | 2 | 0 | 3 | 1 | 14 | 0 | 0 | 19 |
| Unknown | 12 | 6 | 0 | 6 | 1 | 2 | 0 | 4 | 18 |
| t065 | 9 | 6 | 0 | 3 | 0 | 8 | 0 | 1 | 18 |
| t015 | 8 | 4 | 0 | 4 | 0 | 7 | 0 | 2 | 17 |
| t021 | 4 | 2 | 0 | 2 | 0 | 9 | 0 | 1 | 14 |
| t189 | 0 | 0 | 0 | 0 | 0 | 10 | 0 | 4 | 14 |
| t127 | 2 | 1 | 0 | 1 | 0 | 10 | 0 | 0 | 12 |
| t346 | 2 | 1 | 0 | 1 | 0 | 8 | 0 | 0 | 10 |
| t005 | 0 | 0 | 0 | 0 | 0 | 7 | 0 | 2 | 9 |
| t311 | 2 | 1 | 0 | 1 | 0 | 5 | 0 | 2 | 9 |
| t223 | 4 | 3 | 0 | 1 | 0 | 3 | 0 | 1 | 8 |
| t7384 | 0 | 0 | 0 | 0 | 0 | 1 | 0 | 7 | 8 |
| t304 | 2 | 0 | 0 | 1 | 1 | 4 | 1 | 1 | 8 |
| t216 | 1 | 1 | 0 | 0 | 0 | 4 | 0 | 2 | 7 |
| t148 | 1 | 1 | 0 | 0 | 0 | 6 | 0 | 0 | 7 |
| t1451 | 3 | 1 | 0 | 2 | 0 | 4 | 0 | 0 | 7 |
| t166 | 4 | 3 | 0 | 1 | 0 | 2 | 0 | 0 | 6 |
| t190 | 4 | 2 | 0 | 2 | 0 | 2 | 0 | 0 | 6 |
| t094 | 2 | 1 | 0 | 1 | 0 | 4 | 0 | 0 | 6 |
| t309 | 2 | 1 | 0 | 1 | 0 | 3 | 0 | 1 | 6 |
| t085 | 1 | 1 | 0 | 0 | 0 | 3 | 0 | 2 | 6 |
| t267 | 2 | 1 | 0 | 1 | 0 | 2 | 0 | 1 | 5 |
| t701 | 2 | 1 | 0 | 1 | 0 | 2 | 0 | 1 | 5 |
| t837 | 4 | 2 | 0 | 2 | 0 | 1 | 0 | 0 | 5 |
| t408 | 3 | 2 | 0 | 1 | 0 | 2 | 0 | 0 | 5 |
| t362 | 3 | 1 | 0 | 2 | 0 | 2 | 0 | 0 | 5 |
| t024 | 1 | 0 | 0 | 1 | 0 | 2 | 0 | 2 | 5 |
| t1709 | 0 | 0 | 0 | 0 | 0 | 0 | 0 | 5 | 5 |
| t056 | 0 | 0 | 0 | 0 | 0 | 5 | 0 | 0 | 5 |
| t045 | 0 | 0 | 0 | 0 | 0 | 3 | 0 | 1 | 4 |
| t359 | 2 | 1 | 0 | 1 | 0 | 1 | 0 | 1 | 4 |
| t224 | 2 | 1 | 0 | 1 | 0 | 2 | 0 | 0 | 4 |
| t050 | 3 | 2 | 0 | 1 | 0 | 0 | 0 | 1 | 4 |
| t1509 | 2 | 1 | 0 | 1 | 0 | 2 | 0 | 0 | 4 |
| t2734 | 2 | 1 | 0 | 1 | 0 | 2 | 0 | 0 | 4 |
| t364 | 1 | 1 | 0 | 0 | 0 | 3 | 0 | 0 | 4 |
| t19256 | 0 | 0 | 0 | 0 | 0 | 0 | 0 | 4 | 4 |
| t003 | 2 | 1 | 0 | 1 | 0 | 1 | 0 | 0 | 3 |
| t377 | 1 | 0 | 0 | 1 | 1 | 2 | 0 | 0 | 3 |
| t505 | 3 | 2 | 0 | 1 | 0 | 0 | 0 | 0 | 3 |
| t5643 | 3 | 1 | 0 | 2 | 0 | 0 | 0 | 0 | 3 |
| t9038 | 3 | 1 | 0 | 2 | 0 | 0 | 0 | 0 | 3 |
| t605 | 3 | 1 | 0 | 2 | 0 | 0 | 0 | 0 | 3 |
| t2387 | 2 | 2 | 0 | 0 | 0 | 1 | 0 | 0 | 3 |
| t2383 | 2 | 1 | 0 | 1 | 0 | 1 | 0 | 0 | 3 |
| t10686 | 2 | 1 | 0 | 1 | 0 | 1 | 0 | 0 | 3 |
| t2211 | 2 | 1 | 0 | 1 | 0 | 1 | 0 | 0 | 3 |
| t211 | 2 | 1 | 0 | 1 | 0 | 1 | 0 | 0 | 3 |
| t097 | 2 | 1 | 0 | 1 | 0 | 1 | 0 | 0 | 3 |
| t136 | 2 | 1 | 0 | 1 | 0 | 1 | 0 | 0 | 3 |
| t884 | 2 | 1 | 0 | 1 | 0 | 1 | 0 | 0 | 3 |
| t330 | 1 | 1 | 0 | 0 | 0 | 2 | 0 | 0 | 3 |
| t845 | 1 | 1 | 0 | 0 | 0 | 2 | 0 | 0 | 3 |
| t803 | 1 | 0 | 0 | 1 | 0 | 2 | 0 | 0 | 3 |
| t936 | 0 | 0 | 0 | 0 | 0 | 0 | 0 | 3 | 3 |
| t722 | 0 | 0 | 0 | 0 | 0 | 0 | 0 | 3 | 3 |
| t19258 | 0 | 0 | 0 | 0 | 0 | 0 | 0 | 3 | 3 |
| t160 | 0 | 0 | 0 | 0 | 0 | 3 | 0 | 0 | 3 |
| t040 | 0 | 0 | 0 | 0 | 0 | 3 | 0 | 0 | 3 |
| t010 | 1 | 0 | 0 | 1 | 0 | 1 | 0 | 0 | 2 |
| t688 | 0 | 0 | 0 | 0 | 0 | 2 | 0 | 0 | 2 |
| t153 | 0 | 0 | 0 | 0 | 0 | 2 | 0 | 0 | 2 |
| t548 | 1 | 1 | 0 | 0 | 0 | 0 | 0 | 1 | 2 |
| t790 | 0 | 0 | 0 | 0 | 0 | 2 | 0 | 0 | 2 |
| t2413 | 2 | 1 | 0 | 1 | 1 | 0 | 0 | 0 | 2 |
| t156 | 2 | 1 | 0 | 1 | 1 | 0 | 0 | 0 | 2 |
| t19477 | 1 | 0 | 0 | 1 | 1 | 1 | 0 | 0 | 2 |
| t192 | 2 | 1 | 0 | 1 | 0 | 0 | 0 | 0 | 2 |
| t1149 | 2 | 1 | 0 | 1 | 0 | 0 | 0 | 0 | 2 |
| t1406 | 2 | 1 | 0 | 1 | 0 | 0 | 0 | 0 | 2 |
| t268 | 2 | 1 | 0 | 1 | 0 | 0 | 0 | 0 | 2 |
| t4893 | 2 | 1 | 0 | 1 | 0 | 0 | 0 | 0 | 2 |
| t2251 | 2 | 1 | 0 | 1 | 0 | 0 | 0 | 0 | 2 |
| t19450 | 2 | 1 | 0 | 1 | 0 | 0 | 0 | 0 | 2 |
| t11449 | 2 | 1 | 0 | 1 | 0 | 0 | 0 | 0 | 2 |
| t339 | 2 | 1 | 0 | 1 | 0 | 0 | 0 | 0 | 2 |
| t1378 | 2 | 1 | 0 | 1 | 0 | 0 | 0 | 0 | 2 |
| t062 | 2 | 1 | 0 | 1 | 0 | 0 | 0 | 0 | 2 |
| t3959 | 2 | 1 | 0 | 1 | 0 | 0 | 0 | 0 | 2 |
| t067 | 2 | 1 | 0 | 1 | 0 | 0 | 0 | 0 | 2 |
| t19481 | 2 | 1 | 0 | 1 | 0 | 0 | 0 | 0 | 2 |
| t18201 | 2 | 1 | 0 | 1 | 0 | 0 | 0 | 0 | 2 |
| t2427 | 2 | 1 | 0 | 1 | 0 | 0 | 0 | 0 | 2 |
| t19480 | 2 | 1 | 0 | 1 | 0 | 0 | 0 | 0 | 2 |
| t2915 | 2 | 1 | 0 | 1 | 0 | 0 | 0 | 0 | 2 |
| t2930 | 2 | 1 | 0 | 1 | 0 | 0 | 0 | 0 | 2 |
| t279 | 1 | 1 | 0 | 0 | 0 | 1 | 0 | 0 | 2 |
| t289 | 1 | 1 | 0 | 0 | 0 | 1 | 0 | 0 | 2 |
| t2332 | 1 | 1 | 0 | 0 | 0 | 1 | 0 | 0 | 2 |
| t3638 | 1 | 1 | 0 | 0 | 0 | 1 | 0 | 0 | 2 |
| t6605 | 1 | 1 | 0 | 0 | 0 | 1 | 0 | 0 | 2 |
| t728 | 1 | 1 | 0 | 0 | 0 | 1 | 0 | 0 | 2 |
| t8544 | 1 | 1 | 0 | 0 | 0 | 1 | 0 | 0 | 2 |
| t340 | 1 | 1 | 0 | 0 | 0 | 1 | 0 | 0 | 2 |
| t004 | 1 | 1 | 0 | 0 | 0 | 1 | 0 | 0 | 2 |
| t6005 | 1 | 0 | 0 | 1 | 0 | 1 | 0 | 0 | 2 |
| t209 | 1 | 0 | 0 | 1 | 0 | 0 | 0 | 1 | 2 |
| t338 | 0 | 0 | 0 | 0 | 0 | 2 | 0 | 0 | 2 |
| t3508 | 0 | 0 | 0 | 0 | 0 | 2 | 0 | 0 | 2 |
| t131 | 0 | 0 | 0 | 0 | 0 | 2 | 0 | 0 | 2 |
| t1943 | 0 | 0 | 0 | 0 | 0 | 2 | 0 | 0 | 2 |
| t018 | 0 | 0 | 0 | 0 | 0 | 2 | 0 | 0 | 2 |
| t880 | 0 | 0 | 0 | 0 | 0 | 2 | 0 | 0 | 2 |
| t171 | 0 | 0 | 0 | 0 | 0 | 2 | 0 | 0 | 2 |
| t3841 | 0 | 0 | 0 | 0 | 0 | 2 | 0 | 0 | 2 |
| t8099 | 0 | 0 | 0 | 0 | 0 | 2 | 0 | 0 | 2 |
| t11483 | 0 | 0 | 0 | 0 | 0 | 0 | 0 | 2 | 2 |
| t1456 | 0 | 0 | 0 | 0 | 0 | 1 | 0 | 1 | 2 |
| t774 | 0 | 0 | 0 | 0 | 0 | 1 | 0 | 1 | 2 |
| t499 | 0 | 0 | 0 | 0 | 0 | 1 | 0 | 1 | 2 |
| t668 | 1 | 1 | 0 | 0 | 0 | 0 | 0 | 0 | 1 |
| t1510 | 0 | 0 | 0 | 0 | 0 | 1 | 0 | 0 | 1 |
| t630 | 0 | 0 | 0 | 0 | 0 | 1 | 0 | 0 | 1 |
| t179 | 0 | 0 | 0 | 0 | 0 | 1 | 0 | 0 | 1 |
| t442 | 0 | 0 | 0 | 0 | 0 | 1 | 0 | 0 | 1 |
| t4652 | 0 | 0 | 0 | 0 | 0 | 1 | 0 | 0 | 1 |
| t3625 | 0 | 0 | 0 | 0 | 0 | 1 | 0 | 0 | 1 |
| t903 | 0 | 0 | 0 | 0 | 0 | 1 | 0 | 0 | 1 |
| t321 | 0 | 0 | 0 | 0 | 0 | 0 | 0 | 1 | 1 |
| t1034 | 1 | 0 | 0 | 1 | 1 | 0 | 0 | 0 | 1 |
| t300 | 1 | 0 | 0 | 1 | 1 | 0 | 0 | 0 | 1 |
| t8698 | 1 | 0 | 0 | 1 | 1 | 0 | 0 | 0 | 1 |
| t1626 | 1 | 0 | 0 | 1 | 1 | 0 | 0 | 0 | 1 |
| t1636 | 1 | 1 | 0 | 0 | 0 | 0 | 0 | 0 | 1 |
| t12014 | 1 | 1 | 0 | 0 | 0 | 0 | 0 | 0 | 1 |
| t491 | 1 | 1 | 0 | 0 | 0 | 0 | 0 | 0 | 1 |
| t416 | 1 | 1 | 0 | 0 | 0 | 0 | 0 | 0 | 1 |
| t917 | 1 | 1 | 0 | 0 | 0 | 0 | 0 | 0 | 1 |
| t975 | 1 | 1 | 0 | 0 | 0 | 0 | 0 | 0 | 1 |
| t706 | 1 | 1 | 0 | 0 | 0 | 0 | 0 | 0 | 1 |
| t445 | 1 | 1 | 0 | 0 | 0 | 0 | 0 | 0 | 1 |
| t10074 | 1 | 1 | 0 | 0 | 0 | 0 | 0 | 0 | 1 |
| t11639 | 1 | 1 | 0 | 0 | 0 | 0 | 0 | 0 | 1 |
| t4522 | 1 | 1 | 0 | 0 | 0 | 0 | 0 | 0 | 1 |
| t3742 | 1 | 1 | 0 | 0 | 0 | 0 | 0 | 0 | 1 |
| t702 | 1 | 1 | 0 | 0 | 0 | 0 | 0 | 0 | 1 |
| t3546 | 1 | 1 | 0 | 0 | 0 | 0 | 0 | 0 | 1 |
| t587 | 1 | 1 | 0 | 0 | 0 | 0 | 0 | 0 | 1 |
| t2208 | 1 | 0 | 0 | 1 | 0 | 0 | 0 | 0 | 1 |
| t164 | 0 | 0 | 0 | 0 | 0 | 1 | 0 | 0 | 1 |
| t514 | 0 | 0 | 0 | 0 | 0 | 1 | 0 | 0 | 1 |
| t310 | 0 | 0 | 0 | 0 | 0 | 1 | 0 | 0 | 1 |
| t1601 | 0 | 0 | 0 | 0 | 0 | 1 | 0 | 0 | 1 |
| t1937 | 0 | 0 | 0 | 0 | 0 | 1 | 0 | 0 | 1 |
| t780 | 0 | 0 | 0 | 0 | 0 | 1 | 0 | 0 | 1 |
| t9745 | 0 | 0 | 0 | 0 | 0 | 1 | 0 | 0 | 1 |
| t19808 | 0 | 0 | 0 | 0 | 0 | 1 | 0 | 0 | 1 |
| t937 | 0 | 0 | 0 | 0 | 0 | 1 | 0 | 0 | 1 |
| t1238 | 0 | 0 | 0 | 0 | 0 | 1 | 0 | 0 | 1 |
| t177 | 0 | 0 | 0 | 0 | 0 | 1 | 0 | 0 | 1 |
| t19826 | 0 | 0 | 0 | 0 | 0 | 1 | 0 | 0 | 1 |
| t2324 | 0 | 0 | 0 | 0 | 0 | 1 | 0 | 0 | 1 |
| t914 | 0 | 0 | 0 | 0 | 0 | 1 | 0 | 0 | 1 |
| t11249 | 0 | 0 | 0 | 0 | 0 | 1 | 0 | 0 | 1 |
| t2379 | 0 | 0 | 0 | 0 | 0 | 1 | 0 | 0 | 1 |
| t1996 | 0 | 0 | 0 | 0 | 0 | 1 | 0 | 0 | 1 |
| t3266 | 0 | 0 | 0 | 0 | 0 | 1 | 0 | 0 | 1 |
| t19806 | 0 | 0 | 0 | 0 | 0 | 1 | 0 | 0 | 1 |
| t535 | 0 | 0 | 0 | 0 | 0 | 1 | 0 | 0 | 1 |
| t19812 | 0 | 0 | 0 | 0 | 0 | 1 | 0 | 0 | 1 |
| t093 | 0 | 0 | 0 | 0 | 0 | 1 | 0 | 0 | 1 |
| t19814 | 0 | 0 | 0 | 0 | 0 | 1 | 0 | 0 | 1 |
| t19824 | 0 | 0 | 0 | 0 | 0 | 1 | 0 | 0 | 1 |
| t5306 | 0 | 0 | 0 | 0 | 0 | 1 | 0 | 0 | 1 |
| t4389 | 0 | 0 | 0 | 0 | 0 | 1 | 0 | 0 | 1 |
| t318 | 0 | 0 | 0 | 0 | 0 | 1 | 0 | 0 | 1 |
| t2086 | 0 | 0 | 0 | 0 | 0 | 1 | 0 | 0 | 1 |
| t106 | 0 | 0 | 0 | 0 | 0 | 1 | 0 | 0 | 1 |
| t2304 | 0 | 0 | 0 | 0 | 0 | 1 | 0 | 0 | 1 |
| t073 | 0 | 0 | 0 | 0 | 0 | 1 | 0 | 0 | 1 |
| t1170 | 0 | 0 | 0 | 0 | 0 | 1 | 0 | 0 | 1 |
| t1281 | 0 | 0 | 0 | 0 | 0 | 1 | 0 | 0 | 1 |
| t19478 | 0 | 0 | 0 | 0 | 0 | 1 | 0 | 0 | 1 |
| t2171 | 0 | 0 | 0 | 0 | 0 | 1 | 0 | 0 | 1 |
| t913 | 0 | 0 | 0 | 0 | 0 | 1 | 0 | 0 | 1 |
| t1358 | 0 | 0 | 0 | 0 | 0 | 1 | 0 | 0 | 1 |
| t3267 | 0 | 0 | 0 | 0 | 0 | 1 | 0 | 0 | 1 |
| t363 | 0 | 0 | 0 | 0 | 0 | 1 | 0 | 0 | 1 |
| t114 | 0 | 0 | 0 | 0 | 0 | 1 | 0 | 0 | 1 |
| t11580 | 0 | 0 | 0 | 0 | 0 | 1 | 0 | 0 | 1 |
| t272 | 0 | 0 | 0 | 0 | 0 | 1 | 0 | 0 | 1 |
| t2730 | 0 | 0 | 0 | 0 | 0 | 1 | 0 | 0 | 1 |
| t579 | 0 | 0 | 0 | 0 | 0 | 1 | 0 | 0 | 1 |
| t474 | 0 | 0 | 0 | 0 | 0 | 1 | 0 | 0 | 1 |
| t398 | 0 | 0 | 0 | 0 | 0 | 1 | 0 | 0 | 1 |
| t1028 | 0 | 0 | 0 | 0 | 0 | 1 | 0 | 0 | 1 |
| t1508 | 0 | 0 | 0 | 0 | 0 | 1 | 0 | 0 | 1 |
| t1469 | 0 | 0 | 0 | 0 | 0 | 1 | 0 | 0 | 1 |
| t3783 | 0 | 0 | 0 | 0 | 0 | 1 | 0 | 0 | 1 |
| t17524 | 0 | 0 | 0 | 0 | 0 | 1 | 0 | 0 | 1 |
| t360 | 0 | 0 | 0 | 0 | 0 | 1 | 0 | 0 | 1 |
| t9476 | 0 | 0 | 0 | 0 | 0 | 1 | 0 | 0 | 1 |
| t19801 | 0 | 0 | 0 | 0 | 0 | 1 | 0 | 0 | 1 |
| t493 | 0 | 0 | 0 | 0 | 0 | 1 | 0 | 0 | 1 |
| t335 | 0 | 0 | 0 | 0 | 0 | 1 | 0 | 0 | 1 |
| t1070 | 0 | 0 | 0 | 0 | 0 | 1 | 0 | 0 | 1 |
| t731 | 0 | 0 | 0 | 0 | 0 | 1 | 0 | 0 | 1 |
| t723 | 0 | 0 | 0 | 0 | 0 | 1 | 0 | 0 | 1 |
| t280 | 0 | 0 | 0 | 0 | 0 | 1 | 0 | 0 | 1 |
| t242 | 0 | 0 | 0 | 0 | 0 | 1 | 0 | 0 | 1 |
| t3849 | 0 | 0 | 0 | 0 | 0 | 1 | 0 | 0 | 1 |
| t2884 | 0 | 0 | 0 | 0 | 0 | 1 | 0 | 0 | 1 |
| t2787 | 0 | 0 | 0 | 0 | 0 | 1 | 0 | 0 | 1 |
| t582 | 0 | 0 | 0 | 0 | 0 | 1 | 0 | 0 | 1 |
| t2802 | 0 | 0 | 0 | 0 | 0 | 1 | 0 | 0 | 1 |
| t1439 | 0 | 0 | 0 | 0 | 0 | 1 | 0 | 0 | 1 |
| t17678 | 0 | 0 | 0 | 0 | 0 | 1 | 0 | 0 | 1 |
| t064 | 0 | 0 | 0 | 0 | 0 | 1 | 0 | 0 | 1 |
| t068 | 0 | 0 | 0 | 0 | 0 | 1 | 0 | 0 | 1 |
| t071 | 0 | 0 | 0 | 0 | 0 | 1 | 0 | 0 | 1 |
| t2029 | 0 | 0 | 0 | 0 | 0 | 1 | 0 | 0 | 1 |
| t017 | 0 | 0 | 0 | 0 | 0 | 1 | 0 | 0 | 1 |
| t3092 | 0 | 0 | 0 | 0 | 0 | 1 | 0 | 0 | 1 |
| t5695 | 0 | 0 | 0 | 0 | 0 | 1 | 0 | 0 | 1 |
| t1976 | 0 | 0 | 0 | 0 | 0 | 1 | 0 | 0 | 1 |
| t1312 | 0 | 0 | 0 | 0 | 0 | 1 | 0 | 0 | 1 |
| t352 | 0 | 0 | 0 | 0 | 0 | 1 | 0 | 0 | 1 |
| t591 | 0 | 0 | 0 | 0 | 0 | 1 | 0 | 0 | 1 |
| t537 | 0 | 0 | 0 | 0 | 0 | 1 | 0 | 0 | 1 |
| t1627 | 0 | 0 | 0 | 0 | 0 | 1 | 0 | 0 | 1 |
| t4109 | 0 | 0 | 0 | 0 | 0 | 1 | 0 | 0 | 1 |
| t1684 | 0 | 0 | 0 | 0 | 0 | 1 | 0 | 0 | 1 |
| t840 | 0 | 0 | 0 | 0 | 0 | 1 | 0 | 0 | 1 |
| t10721 | 0 | 0 | 0 | 0 | 0 | 1 | 0 | 0 | 1 |
| t9210 | 0 | 0 | 0 | 0 | 0 | 1 | 0 | 0 | 1 |
| t12618 | 0 | 0 | 0 | 0 | 0 | 1 | 0 | 0 | 1 |
| t19810 | 0 | 0 | 0 | 0 | 0 | 1 | 0 | 0 | 1 |
| t2518 | 0 | 0 | 0 | 0 | 0 | 1 | 0 | 0 | 1 |
| t1172 | 0 | 0 | 0 | 0 | 0 | 1 | 0 | 0 | 1 |
| t369 | 0 | 0 | 0 | 0 | 0 | 1 | 0 | 0 | 1 |
| t19815 | 0 | 0 | 0 | 0 | 0 | 1 | 0 | 0 | 1 |
| t3262 | 0 | 0 | 0 | 0 | 0 | 1 | 0 | 0 | 1 |
| t15707 | 0 | 0 | 0 | 0 | 0 | 1 | 0 | 0 | 1 |
| t213 | 0 | 0 | 0 | 0 | 0 | 1 | 0 | 0 | 1 |
| t1908 | 0 | 0 | 0 | 0 | 0 | 1 | 0 | 0 | 1 |
| t031 | 0 | 0 | 0 | 0 | 0 | 1 | 0 | 0 | 1 |
| t1228 | 0 | 0 | 0 | 0 | 0 | 1 | 0 | 0 | 1 |
| t19811 | 0 | 0 | 0 | 0 | 0 | 1 | 0 | 0 | 1 |
| t1399 | 0 | 0 | 0 | 0 | 0 | 1 | 0 | 0 | 1 |
| t19803 | 0 | 0 | 0 | 0 | 0 | 1 | 0 | 0 | 1 |
| t306 | 0 | 0 | 0 | 0 | 0 | 1 | 0 | 0 | 1 |
| t777 | 0 | 0 | 0 | 0 | 0 | 1 | 0 | 0 | 1 |
| t1135 | 0 | 0 | 0 | 0 | 0 | 1 | 0 | 0 | 1 |
| t275 | 0 | 0 | 0 | 0 | 0 | 1 | 0 | 0 | 1 |
| t19823 | 0 | 0 | 0 | 0 | 0 | 1 | 0 | 0 | 1 |
| t1255 | 0 | 0 | 0 | 0 | 0 | 1 | 0 | 0 | 1 |
| t10321 | 0 | 0 | 0 | 0 | 0 | 1 | 0 | 0 | 1 |
| t2833 | 0 | 0 | 0 | 0 | 0 | 1 | 0 | 0 | 1 |
| t6197 | 0 | 0 | 0 | 0 | 0 | 1 | 0 | 0 | 1 |
| t19804 | 0 | 0 | 0 | 0 | 0 | 1 | 0 | 0 | 1 |
| t328 | 0 | 0 | 0 | 0 | 0 | 1 | 0 | 0 | 1 |
| t1412 | 0 | 0 | 0 | 0 | 0 | 1 | 0 | 0 | 1 |
| t324 | 0 | 0 | 0 | 0 | 0 | 1 | 0 | 0 | 1 |
| t1875 | 0 | 0 | 0 | 0 | 0 | 1 | 0 | 0 | 1 |
| t1350 | 0 | 0 | 0 | 0 | 0 | 1 | 0 | 0 | 1 |
| t2409 | 0 | 0 | 0 | 0 | 0 | 1 | 0 | 0 | 1 |
| t1126 | 0 | 0 | 0 | 0 | 0 | 1 | 0 | 0 | 1 |
| t3698 | 0 | 0 | 0 | 0 | 0 | 1 | 0 | 0 | 1 |
| t19819 | 0 | 0 | 0 | 0 | 0 | 1 | 0 | 0 | 1 |
| t19813 | 0 | 0 | 0 | 0 | 0 | 1 | 0 | 0 | 1 |
| t948 | 0 | 0 | 0 | 0 | 0 | 1 | 0 | 0 | 1 |
| t5615 | 0 | 0 | 0 | 0 | 0 | 1 | 0 | 0 | 1 |
| t10984 | 0 | 0 | 0 | 0 | 0 | 1 | 0 | 0 | 1 |
| t6910 | 0 | 0 | 0 | 0 | 0 | 1 | 0 | 0 | 1 |
| t214 | 0 | 0 | 0 | 0 | 0 | 0 | 0 | 1 | 1 |
| t282 | 0 | 0 | 0 | 0 | 0 | 0 | 0 | 1 | 1 |
| t2201 | 0 | 0 | 0 | 0 | 0 | 0 | 0 | 1 | 1 |
| t8189 | 0 | 0 | 0 | 0 | 0 | 0 | 0 | 1 | 1 |
| t2558 | 0 | 0 | 0 | 0 | 0 | 0 | 0 | 1 | 1 |
| t19821 | 0 | 0 | 0 | 0 | 0 | 0 | 0 | 1 | 1 |
| t19807 | 0 | 0 | 0 | 0 | 0 | 0 | 0 | 1 | 1 |
