# Supplementary material for: Dynamics of Staphylococcus aureus in patients and the hospital environment in a tertiary care hospital in the Netherlands
Source: Antimicrob Resist Infect Control. 2023 Dec 20;12:148. doi: 10.1186/s13756-023-01349-2 (PMC10734193; doi:10.1186/s13756-023-01349-2)
Supplement: Supplementary file 4 — Supplementary Material 4: Positive Staphylococcus aureus locations. [file 13756_2023_1349_MOESM4_ESM.docx]

**Supplementary file 4. Positive *Staphylococcus aureus* locations**

**Supplementary table 4.1** MSSA identified on locations in the old and the new hospital building.

|  | **Old hospital building** |  |  | **New hospital building** |  |
| --- | --- | --- | --- | --- | --- |
| **Room type** | **Location** | **MSSA (%)** | **Room type** | **Location** | **MSSA (%)** |
| Two- and four patient room | Nightstand (n=149) | 8 (5.4) | Single-occupancy room | Nightstand (n=315) | 14 (4.4) |
|  | Table (n=79) | 4 (5.1) |  | Table (n=324) | 16 (4.9) |
|  | Wall (n=79) | - |  | Wall (n=324) | 3 (0.9) |
|  | Sink (n=50) | 1 (2.0) |  | Sink (n=324) | 9 (2.8) |
|  | Top of sink plug (n=50) | 2 (4.0) |  | Top of sink plug (n=324) | 8 (2.5) |
|  | Bottom of sink plug (n=20) | 1 (5.0) |  | Bottom of sink plug (n=322) | 3 (0.9) |
| ICU room | Nightstand (n=20) | - | ICU room | Nightstand (n=128) | 7 (5.5) |
|  | Wall (n=20) | - |  | Wall (n=150) | 2 (1.3) |
|  | Sink (n=24) | - |  | Sink (n=181) | 2 (1.1) |
|  | Top of sink plug (n=24) | 1 (4.2) |  | Top of sink plug (n=181) | 4 (2.2) |
|  | Bottom of sink plug (n=24) | - |  | Bottom of sink plug (n=181) | 4 (2.2) |
| Room with anteroom | Nightstand (n=14) | - | Room with anteroom | Nightstand (n=88) | 3 (3.4) |
|  | Table (n=14) | - |  | Table (n=93) | 2 (2.2) |
|  | Wall (n=14) | - |  | Wall (n=95) | - |
|  | Sink (n=14) | 1 (7.1) |  | Sink (n=95) | 1 (1.1) |
|  | Top of sink plug (n=14) | - |  | Top of sink plug (n=95) | 1 (1.1) |
|  | Bottom of sink plug (n=0) | - |  | Bottom of sink plug (n=95) | 1 (1.1) |
| Bathroom | Toilet seat (n=17) | 1 (5.9) | Private bathroom | Toilet seat (n=138) | 5 (3.6) |
|  | Shower chair (n=17) | - |  | Shower chair (n=138) | 3 (2.2) |
|  | Shower drain (n=17) | 2 (11.8) |  | Shower drain (n=138) | 19 (13.8) |
|  | Door handle (n=18) | 1 (5.6) |  | Door handle (n=138) | 3 (2.2) |
|  | Sink (n=20) | - |  | Sink (n=138) | 3 (2.2) |
|  | Top of sink plug (n=20) | - |  | Top of sink plug (n=138) | 4 (2.9) |
|  | Bottom of sink plug (n=4) | - |  | Bottom of sink plug (n=126) | 3 (2.4) |

Abbreviations: MSSA methicillin sensitive *Staphylococcus aureus*.

**Supplementary table 4.2** Percentages of MSSA positive locations per sampling moment.

|  | Sampling moment | Percentage of MSSA positive locations |
| --- | --- | --- |
| Old building | April 2018 | 1.6% |
|  | May 2018 | 4.4% |
| New building | May 2018 (two weeks before) | 0.7% |
|  | May 2018 (one week before) | 2.0% |
|  | May 2018 (one day before) | 0.5% |
|  | May 2018 (one day after) | 2.7% |
|  | May 2018 (one week after) | 3.6% |
|  | June 2018 (two weeks after) | 2.3% |
|  | June 2018 | 2.0% |
|  | August 2018 | 2.7% |
|  | November 2018 | 4.6% |
|  | February 2019 | 8.5% |
|  | May 2019 | 1.3% |
|  | August 2019 | 1.6% |
|  | November 2019 | 5.6% |
|  | May 2020 | 2.3% |
|  | May 2021 | 0.7% |
